# Assessment of hepatitis B knowledge and awareness among the Sudanese population in Khartoum State

**DOI:** 10.11604/pamj.2022.41.217.30390

**Published:** 2022-03-16

**Authors:** Omer Osman Kheir, Catherine Freeland, Abdelmounem Eltayeib Abdo, Mortada Elrashid Mohamed Yousif, Eilaf Osama Altayeb, Hailemichael Desalegn Mekonnen

**Affiliations:** 1 Research Unit, National Center for Gastrointestinal and Liver Diseases (NCGLD), Khartoum, Sudan,; 2 Hepatitis B Foundation, 3805 Old Easton Rd., Doylestown, PA 18902 USA,; 3 Gastroenterology Sub-Saharan Africa ECHO St. Paul´s Hospital Millennium Medical College, Addis Ababa, Ethiopia

**Keywords:** Hepatitis B virus, global public health, liver disease, Sudan

## Abstract

**Introduction:**

globally it is estimated that majority of the burden of hepatitis B virus infection is in sub-Saharan African countries (SSA). Africa is also hit by a dreadful complication of hepatocellular carcinoma and sequalae of end-stage liver disease. Despite this, the knowledge and awareness of the population to this silent killer is largely unknown. The aim of this study was to assess the knowledge and awareness of hepatitis B virus among the general population within Sudan to understand the misconceptions and provide a better direction toward the disease elimination goals.

**Methods:**

a community-based study was carried out in three locations in Khartoum state during a community hepatitis awareness campaign, where participants were provided education, screening, and vaccine. Data were collected after proper consent was obtained from the respective Institutional Review Board (IRB) office. Basic demographic characteristics, knowledge assessment questions, and awareness were used, which are derived from standard questionnaire. Finally, basic descriptive statistics were undergone to assess the knowledge and awareness of the participants.

**Results:**

the study has shown that self-reported hepatitis B among the participants was 9.6%. There are areas of hepatitis B misconception in knowledge and awareness related to transmission, modes of prevention and disease state. We have also noticed that prior vaccine coverage was low among the groups, which is also another major concern.

**Conclusion:**

the prevalence of hepatitis B from these randomly selected population groups is high. There is also lower vaccine coverage and many misconceptions in knowledge and awareness of hepatitis B. Policymakers should consider these issues seriously to improve the gaps in hepatitis B.

## Introduction

Viral hepatitis caused 1.34 million deaths in 2015, mostly due to chronic liver disease (720 000 deaths due to cirrhosis) and primary liver cancer (470 000 deaths due to hepatocellular carcinoma (HCC)) [1]. Globally, an estimated 290 million people are living with chronic hepatitis B infection, which is responsible for a large proportion of hepatitis mortality and morbidity. Sub-Saharan Africa has one of the highest hepatitis B-related liver cancer rates worldwide [2-4]. Globally, it is estimated that 21 countries account for 80% of the total burden of hepatitis B infections within the general population. These countries are found within sub-Saharan Africa (SSA), Asia and the Pacific [5]. Despite these alarming estimates, testing for hepatitis B remains low, even among those considered high-risk [6, 7]. In response to the United Nations Sustainable Development Goals, which aim to eliminate viral hepatitis as a public health threat by the year 2030 (2016-2021), hepatitis B infection is receiving increasing recognition with efforts to upscale prevention, diagnosis, and treatment [8]. The World Health Organization (WHO) launched an ambitious goal in 2015 to reduce hepatitis B infections by 90% and increase global vaccine coverage to 90% [1]. To reach global goals, health education is an important tool that can improve sensitization for hepatitis B prevention, screening, and vaccination [8].

Over 90% of persons are unaware of their hepatitis B infection, and remain undiagnosed, unmanaged and at risk for cirrhosis, and HCC [9]. This high mortality and morbidity are due in part to the fact that those infected often are asymptomatic with the virus for up to 3 decades; therefore, testing is mostly conducted when liver damage is already severe. Moreover, there is a general lack of awareness within the populations at risk, with studies showing average to poor knowledge of hepatitis B virus infection and low coverage of hepatitis vaccine among persons living in regions of high prevalence [10]. Within the African continent, where hepatitis B is endemic, sensitization campaigns are needed to improve overall awareness, testing, and prevention of hepatitis B. Global research has shown that most populations in endemic areas have poor awareness of hepatitis B and hepatitis C modes of transmission, signs and symptoms, complications, and treatment modalities despite high prevalence of infections, this in turn leads to ineffective preventive measures [11, 12]. Previous research within Africa demonstrates that in Sudan and Morocco, for example, most of the health care workers had basic knowledge of blood as a medium of infection but lacked vaccine coverage [13]. While research has been conducted within the context of health care workers, there is limited information on the knowledge level of the general population within Sudan. This study sought to assess the knowledge and awareness of hepatitis B virus among the general population within Khartoum State Sudan to highlight areas of knowledge gaps and misconceptions that can inform tailored public health sensitization campaigns in the future.

## Methods

**Study settings:** this community based cross-sectional study was carried out in three locations in Khartoum state (Bet al mal social club, Alsaha garden and Al Zahra social club) using a convenience sample.

**Selection of study participants:** eligible participants were greater than 18 years old and agreed to participate. The data was collected by three physicians during a community hepatitis awareness campaign that provided screening and vaccination, along with educational sessions (November 2019- November 2020). The sample was determined as 197 participants with a margin of error of 5% assuming an average anticipated prevalence of hepatitis B as 6.8% [14].

**Data collection procedures:** the questionnaire composed of four sections, demographic characteristics of the community followed by an assessment of self-reported screening and vaccination status of the participants and the third section assessed hepatitis B knowledge (including symptoms, transmission, basic biological characteristics of hepatitis B). The knowledge assessment included 24 questions with yes or no responses and scored with the correct answer was given 1 point and wrong answers were given 0 points, with a total possible maximum score of 24. The final section involved hepatitis awareness related question´s questionnaire was rated by ‘yes’ or ‘no’ or ‘I don´t know’, correct answer was given 1 and wrong answers was given 0, with total possible minimum score of 0 and maximum score of 7. The knowledge and awareness questions were utilizes and based on previous surveys with similar objectives [15-17]. Those who completed the questionnaire were provided an educational session. A week later, participants were contacted by email to complete the questionnaire that involved only the knowledge and awareness questions. The questionnaire was translated into Arabic language by a bilingual translator with forward and backward translation for linguistic validity.

**Statistical analysis:** the data was analyzed using SPSS statistical software. Categorical data was presented in percentages and frequencies, continuous data was presented in mean and standard deviations.

**Ethical consideration:** an ethical clearance was secured and obtained from national center for gastrointestinal and liver diseases; ensuring adherence to ethical principles such as specified by the World Medical Association Declaration of Helsinki. Each participant signed an informed consent after a detailed explanation of the study. The participants were assured anonymity and confidentiality.

## Results

### Demographic characteristics

A total of 197 people has responded in the survey from a total of 225 questionnaire distributed, making the response rate 87.5%. The age distribution showed most are in the age group of 14-37 years of 151 (76.6%) of participants, with a mean age of 28.9±16.3 years. Male had higher representation with 152 (77.2%). One hundred thirty-nine (70.6%) were single, most of the participants 87 (44.2%) were students. The majority (89.3%) of participants reported no previous hepatitis B vaccine. The hepatitis B positivity from the participants was 19 (9.6%) (Table 1).

**Table 1:** characteristics of the study respondents (N = 197)

Characteristics	Number	Percent
**Age**		
14-37	151	76.6
38-57	29	14.7
58-67	9	4.6
68+	8	4.1
**Gender**		
Male	152	77.2
Female	45	22.8
**Job**		
Public sector	20	10.2
Private sector	26	13.2
Self-employed	23	11.7
Student	87	44.2
Retired	7	3.6
Unemployed	34	17.3
**Educational attainment**		
Never been to school	25	12.7
Primary school	57	28.9
High school	36	18.3
Diploma/certificate	15	7.6
Undergraduate/university	29	14.7
Postgraduate	35	17.8
**House hold monthly income**		
Low (1-10000)	128	65.0
Middle (10,001-20,000)	48	24.4
High (>20,000)	21	10.7
**Previous HBV vaccine**		
No	176	89.3
Yes	20	10.2
**Family HBV exposure**		
No	156	79.2
Yes	14	7.1
I don´t know	27	13.7
History of surgery		
No	153	77.7
Yes	44	22.3
**History of blood transfusion**		
No	185	93.9
Yes	12	6.1
**History of blood donation**		
No	153	77.7
Yes	44	22.3
HBsAg		
Negative	178	90.4
Positive	19	9.6

### Assessment of knowledge towards hepatitis B

Knowledge was assessed by questions focusing on hepatitis B etiology, signs and symptoms, transmission, treatment, and management. Each response was scored as ‘yes’ or ‘no’. The scoring range of the questionnaire was 21 (maximum) to 0 (minimum). The participants scored a minimum of 7 and one participant scored the higher score of 21. The mean score of knowledge was 14.1 and values higher than the mean were considered to have good knowledge. Knowledge scores for individuals were calculated and summed up to give the total knowledge score. The knowledge score has shown that 111 (56.3) have poor knowledge and 86 (43.7) have good knowledge about hepatitis B virus (HBV) infection. Poor knowledge was found in hepatitis B infection effect on other systems like brain, kidney, heart. In the hepatitis B transmission part, nearly one-third did not have knowledge of maternal to child transmission, and a higher number have poor knowledge about the transmission through tattoo, unprotected sex (Table 2).

**Table 2:** responses to hepatitis B knowledge items

Hepatitis B knowledge items	Yes number (percent)	No number (percent)
Hepatitis caused by virus	144 (73.1)	53 (26.9)
Brain can be affected by hepatitis B infection	89 (45.2)	108 (54.8)
Kidneys can be affected by hepatitis B infection	130(66.0)	67 (34.0)
Heart can be affected by hepatitis B infection	111 (56.3)	86 (43.7)
Hepatitis B can be transmitted through eating with or sharing food/utensils	102 (51.8%)	94 (48.2)
Hepatitis B can be transmitted through contaminated water	129 (61.4)	68 (34.5)
Hepatitis B can be transmitted through sneezing or coughing	121 (61.4)	76 (38.6)
Hepatitis B can be transmitted through blood	166 (84.3)	31 (15.7)
Hepatitis B can be transmitted through tattoos	122 (61.9)	75 (38.1)
Hepatitis B can be transmitted through unprotected sex	126 (64.0)	71 (36.0)
Hepatitis B can be transmitted through sharing needles	167 (84.8)	30 (15.2)
Hepatitis B can be spread through infected mother to infant hepatitis B during pregnancy	142 (72.1)	55 (27.9)
Hepatitis B can be spread by shaking hands with an infected person	50 (25.4)	147 (74.6)
Hepatitis B infection can be prevented with a vaccine against hepatitis B	163 (82.7)	34 (17.3)
Hepatitis B infection can be prevented with exercise	99 (50.3)	98 (49.7)
Hepatitis B infection can be prevented with a balanced diet	133 (67.5)	64 (32.5)
Hepatitis B infection can be prevented with good hand hygiene	151 (76.6)	46 (23.4)
There is a blood test to detect hepatitis B infection	166 (84.3)	31 (15.7)
There are antiviral therapy for hepatitis B	144 (73.1)	53 (26.9)
**Hepatitis B is a risk factor for liver cancer**	149 (75.6)	48 (24.4)
Hepatitis B infection can be transmitted to your partner	143 (72.6)	54 (27.4)
Newborns must take hepatitis B vaccine	104 (52.8)	93 (47.2)
A complete set of hepatitis B vaccine requires three injections of vaccines	109 (55.3)	88 (44.7)
Jaundice is one of the most common signs of hepatitis B infection	119 (60.4)	78 (39.6)

### Assessment of awareness towards hepatitis B

Awareness towards hepatitis B was assessed by asking seven questions, as shown in Table 3. Each question was labelled with a yes, no and do not know responses. A score of 1 was given to positive while 0 was given to negative attitudes with a score range of maximum of 7 to a minimum of 0. The mean value for awareness was calculated and was found to be 3.05 +/- 1.48 (2-4). A value above the mean was considered a good awareness, and a level below that as a poor awareness. Nearly half of the patients do not know their HBV status (50.8%), only very few 24 (12.2%) have completed hepatitis B vaccination. One hundred eighteen (59.6%) of the participants do not know whether hepatitis B infection remains for the rest of their life. Over all the respondents had a negative awareness towards hepatitis B with mean score of 3.05± 1.4 (Table 3).

**Table 3:** responses to hepatitis B awareness items

N^o^	Question	Yes Number (%)	No Number (%)	Do not know Number (%)
1	Government provides free HB vaccination for infants in Sudan	106 (53.8)	73 (37.1)	18 (9.1)
2	I know the status of my family members Hepatitis B	99 (50.3)	53 (26.9)	45 (22.8)
3	I know my status Hepatitis B	97 (49.2)	57 (28.9)	43 (21.8)
4	I have complete Hepatitis B vaccination	24 (12.2.)	168 (85.3)	5 (2.5)
5	If you received vaccination, you do not need a screening test	96 (48.7)	88 (44.7)	12 (6.1)
6	All the individuals infected with HBV remain infected for the rest of their life	79 (40.1)	99 (50.3)	19 (9.6)
7	Do you know if you are immune, infected with HBV, or at risk?	74 (37.6)	63 (32.0)	60 (30.5)

## Discussion

Study findings demonstrate the need for sensitization campaigns to address reported misconceptions. These findings are similar to what has been found in other countries related to knowledge and awareness of hepatitis B transmission and prevention, but this is one of the first to our knowledge conducted in Sudan. Seroprevalence of hepatitis B surface antigen (HBsAg) has been demonstrated in previous research ranging from 6.8% in central Sudan to as high as 26% in southern Sudan [14]. Our study sample had a self-reported HBsAg prevalence of 9.67% which is considerably high. While this is a self-reported metric, it is still significant. Identified risk factors for infection in Sudan include parenteral anti-schistosomal therapy, sexual transmission, scarification, and perinatal transmission. The prevalence rates reported in Sudan are comparable to other African countries). Previous research has documented improvement strategies to addressing hepatitis B within Sudan including the screening of blood and blood products in blood banks, and extended vaccination in country, however, based on prevalence rates, more work should be done to address hepatitis B in those living with the disease including expansion of access to treatment and management at the provider level [14]. It is also important to note the importance of adding to the body of research in Sudan related to viral hepatitis and the need for research and resources to prevent the spread of infection, promote prevention efforts and increase sensitization among the general public.

Within this study, survey participants reported a lack of previous vaccination to protect against hepatitis B, which is of concern, particularly because this population is considered high risk. According to the World Health Organization, persons within endemic areas of the world should be vaccinated against hepatitis B. The sample population comes from Sudan, which is considered an endemic country with a population prevalence greater than 2% [18, 19]. To address hepatitis B and work towards World Health Organization goals of viral hepatitis elimination which includes reduction in incidence of hepatitis B infections and their associated mortality by 2030, comprehensive public health efforts should work to continue to support education, screening and vaccination at the community level (by 90% and 65% respectively) [19]. An essential public health prevention strategy should work towards directly addressing these targets, including improving vaccination coverage within this region. This involves working to make the vaccine more accessible, available in places of convenience and affordable. The hepatitis B vaccination recommendations within Sudan and other endemic countries should promote vaccination against hepatitis B for all individuals residing in the country and work towards universal vaccination coverage. Additionally, birth dose vaccination administration within the first 24 hours of birth should be administered universally to prevent transmission from happening from mother to child. Implementing hepatitis B birth dose alone has the ability to prevent up to 90% of mother to child transmission of hepatitis B [2, 20].

Based on participant responses, there is a significant knowledge gap in terms of hepatitis B related misconceptions, routes of hepatitis B transmission and what body parts are impacted by hepatitis B. Many individuals (45%, 56% and 66% respectively) reported that the brain, kidney, and heart are affected by a hepatitis B infection. Additionally, many correctly reported that hepatitis B could be transmitted through blood (85%) however, there were significant misconceptions regarding casual contact transmission which has been reported within the literature in other countries [21, 22]. Within this study, 75% falsely reported hepatitis B being spread by shaking hands with someone infected. While the blood transmission, tattoo, unclean needles were reported correctly by most participants, there is still a misconception related to casual contact. Participants were asked if hepatitis B can be prevented with exercise, and over half reported that it could. Similarly, almost 60% reported that a balanced diet can also prevent hepatitis B infection. Both misconceptions are false, and it is important that individuals are readily aware of the most effective mode of hepatitis B prevention through the three-dose hepatitis B vaccination. In addition, sensitization should also work to improve vaccination knowledge, from the study, only 55% were aware that the hepatitis B vaccine was taken in three doses to ensure full protection against the virus.

Ultimately, while this study was effective at identifying areas of need for future research and public health programming related to hepatitis B, there are study limitations that should be noted. First, the population has limited external validity and generalization because the population was a convivence sample of mostly younger (14-37 year), males who identified as single. Future studies should ensure that the population is well representative of the general Sudanese population and include females and a more diverse age range and varied marital status that represents the general population characteristics. Another limitation is the study location, from the Sudanese public health epidemiological data we know that some regions of Sudan have higher prevalence of hepatitis B compared to others. The volume of hepatitis B cases, might impact the community´s overall knowledge levels related to hepatitis B. It is important that additional research should examine knowledge levels throughout Sudan. The reliability test of the questionnaire was not performed could be considered as potential limitation.

Overall, it is clear that there is a lack of information and high prevalence of hepatitis B within Sudan. The World Health Organization and leading medical bodies recognize the global burden of hepatitis B and has called for the elimination of hepatitis B by 2030 [19]. With 60 million estimated hepatitis B infections in Africa public health efforts must be specific to address barriers within Sudan, which has communities that are disproportionately affected by hepatitis B.

## Conclusion

The results of this study contribute valuable information to the currently limited literature documenting the current knowledge level on hepatitis B treatment, prevention and symptoms within this community and can serve as a starting point to inform culturally relevant public health interventions aimed at increasing hepatitis B knowledge and awareness, screening, and vaccination efforts in Sudan. More research is needed to better understand some of the potential barriers to accessing testing and vaccination within this context, and more funding and support to address sensitization at the public health level for hepatitis B awareness and testing is a significant need in this community.

### What is known about this topic

- Research has been conducted within the context of health care workers including nurses, midwives, medical laboratory students and blood donors;
- There is limited information on the HBV vaccination status among general population;
- There is limited information on the knowledge level of the general population within Sudan.

### What this study adds

- Contribute valuable information to the currently limited literature documenting the current knowledge level on hepatitis B treatment, prevention and symptoms within this community; fifty six percent showed poor knowledge about HBV infections;
- Eighty-nine percent reported no previous hepatitis B vaccine;
- Seroprevalence of hepatitis B surface antigen (HBsAg) was 9.67%.
